# A Persons-Centered Approach for Prevention of COVID-19 Disease and Its Impacts in Persons With Disabilities

**DOI:** 10.3389/fpubh.2020.608958

**Published:** 2021-02-05

**Authors:** Suraj Singh Senjam

**Affiliations:** Department of Community Ophthalmology, Dr. Rajendra Prasad Centre for Ophthalmic Sciences, All India Institute of Medical Sciences, New Delhi, India

**Keywords:** the COVID-19, impact on persons with disabilities, preventive strategies, person-centered analysis/approach, international classification for functioning health and disability

## Abstract

The World Health Organization (WHO) considers COVID-19 a great threat to humanity and, thus, declared the COVID-19 outbreak a pandemic on March 11, 2020. To limit its transmission, governments announced lockdowns in their respective nations, and recommended control measures, including behavior change. Persons with disabilities (PwDs) are among the population that may be at a higher risk of becoming infected and may suffer serious illness due to COVID-19. Additionally, lockdowns pose immense challenges and have tremendous impacts on PwDs in terms of receiving their daily support. To mitigate these challenges, their impact, and to reduce the risk of infection, it is important to design strategies that can improve the overall outcome for PwDs. This study therefore intends to provide a uniform strategy or guideline using the person-centered approach principles which is perhaps the most feasible and implementable approach to circumvent the challenges faced by PwDs during emergency lockdowns. Two case studies are used as examples. This pandemic also provides an opportunity for health care planners and policymakers in the health sector to implement reforms to ensure disability inclusiveness in potential future emergency lockdowns.

## Introduction

In late December 2019, the deadly novel coronavirus disease (COVID-19) caused by severe acute respiratory syndrome coronavirus 2 (SARS-CoV-2) was identified in Wuhan, China (1, 2). Shortly after its identification, the disease spread rapidly across the world, leading to a number of deaths. Since COVID-19 has become a severe global health crisis, the WHO officially declared it a pandemic on March 11, 2020. As of September 29, 2020, the total confirmed cases of COVID-19 have reached more than 33 million, with over a million deaths worldwide (3). The SARS-CoV-2 is transmitted from one person to another, primarily through respiratory droplets or direct contact with contaminated hands or surfaces and then by touching the eyes, nose, and mouth. The first reported transmission however, occurred from wild animals to humans (4). The typical common symptoms of COVID-19 include a fever, coughing, and shortness of breath. Other reported symptoms, though rare, include a sore throat, fatigue, and muscle pain.

As part of intervention and control measures, governments across the world imposed lockdowns in their respective countries where all transport, including airports, was shut down; and all shops, markets, and schools were closed. Later, a community containment measure with a strict prohibition of movement in hot-spot areas was carried out. Further, additional mitigation strategies were implemented, including behavior change such as wearing of face masks, observing social or physical distance, frequent handwashing and so on. As of September 29, 2020, no medicine for treatment or a vaccine for prevention is available in the world (5).

During the pandemic and emergency circumstances, everyone faces problems or crises, but crises faced by PwDs might be more severe than those faced by any other individual. In such a situation, a set of measures, involving skilled and unskilled manpower, needs to be adopted to ensure care or services to PwDs. There is a need to develop a support system, using universal and inclusive approaches, to care for these special vulnerable groups of people during a lockdown. Researchers across the world have also recommended that there be a disability -inclusive response when addressing the COVID-19 pandemic (6–8).

This current article attempts to present the potential impact of the COVID-19 pandemic and emergency lockdown on PwDs and provides a universal framework or guideline that can readily be considered while planning for care during such a circumstance, irrespective of the different types of disabilities. It applies the principles of the Person Centered Approach (PCA) which requires a group of individuals (Core Groups) for implementation.

## Disability and the COVID-19 Pandemic

PwDs are at a higher risk of being impacted by both the pandemic and the measures being taken to control the pandemic (7, 9). In addition, they may be at a higher risk of becoming infected with SARS-CoV-2 than people without disabilities (10). There are several reasons that may contribute to this higher risk of infection. This could include a lack of adequate knowledge about COVID-19, attributed to the absence of accessible formats of information, frequent need of personal assistance among PwDs leading to increased exposure, inadequate knowledge of preventive strategies like wearing of face masks and handwashing techniques, and poor practices of the disinfection of their assistive devices. For example, a person with a visual impairment relies on touch for daily living activities and mobility, which may increase the chance of infection. Other factors can be attributed to an inaccessible physical environment and infrastructure, and poor accessibility to health care facilities. They are also at risk of negative impacts resulting from the response to the pandemic, due to interruption of daily supplies, closure of out-patient departments of healthcare institutes, and suspension of transportation which impedes caregiver commutes.

In addition, PwDs may have a higher risk of premature death than those without disabilities because of co-morbidities or existing health conditions. In general, PwDs may have poorer health and are vulnerable to secondary medical problems, such as heart problems, diabetes, or respiratory illness (11). For instance, people with a spinal cord injury, may develop urinary tract infection (related to disability), or PwDs may likely develop heart diseases along with complications like diabetes or hypertension, or PwDs may be at risk of contracting flu (12). Furthermore, premature death among PwDs could be due to the absence of a caregiver during the pandemic. In China, a disabled teenager who was left alone died when his relatives and caregivers were in quarantine (13).

PwDs are also more likely to be excluded from schools and have an incomplete education compared to mainstream students. The COVID-19 pandemic followed by the closure of education institutions, will likely further exacerbate the negative impact of education among PwDs. Further, the widespread use of virtual education may not be feasible for students with visual impairments (14). A study reported that a large number of students in Bangladesh are suffering from depression and anxiety due to the pandemic and lockdown (15). Such psycho-social stress may also cause a disproportionate impact among PwDs living both in the community and in institutions like day care centers, in hostel facilities of schools, rehabilitation centers, and vocational training centers. For example, there were reports of panic, stress, and anxiety felt among visually challenged students staying at hostel facilities at schools for the blind, as teachers and other staff were absent due to the sudden lockdown in Delhi (Vision Rehabilitation Staff Dr. Rajendra Prasad Centre for Ophthalmic Sciences, New Delhi, personal communication, October 20, 2020). Evidence also exists that the number of deaths increased from 42 to 57% in care homes in some countries (16, 17).

The pandemic has also led to a massive disruption in the labor market, resulting in a huge economic crisis among many households, including in households with PwDs. An estimate of the impact on global poverty shows that the number of people living in poverty might increase by almost 500 million from the figures reported in 2018. Such strains on economies and livelihoods will, in particular, be much higher in low-and middle-income countries and for PwDs (18). For example, a survey conducted during the pandemic in Bangladesh reported an income drop of 75% in urban areas and 62% in rural areas and many people are facing livelihood uncertainty (19). Further studies on the impact on various types of disabilities, not only health impacts, but also other psychological and psychosocial impacts, and impacts on support systems during the pandemic, are of great importance.

## Magnitude of Disabilities

The WHO estimates that more than 1 billion people (15% of the world population) live with some degree of disability, and nearly 80% of these people come from low middle-income countries, including India (20). For example, the *World Report on Disability* shows that the prevalence of disability is 24.9% in India. Various regional studies in India reported that the prevalence of disability ranges from 2.02 to 64% (21–23). Empirical studies show that the disabled population, particularly from low middle-income countries, have decreased access to health care services, are often isolated, suffer from poor hygiene, sanitation, and malnutrition, are frequently associated with poverty and poor living conditions, and face an increased risk of additional health problems (24, 25). Although this population has the same health care needs as those of people without disabilities, they experience various hindrances or barriers in accessing, and meeting their health care needs.

## The ICF-WHO Framework of Disability

**Figure 1:**
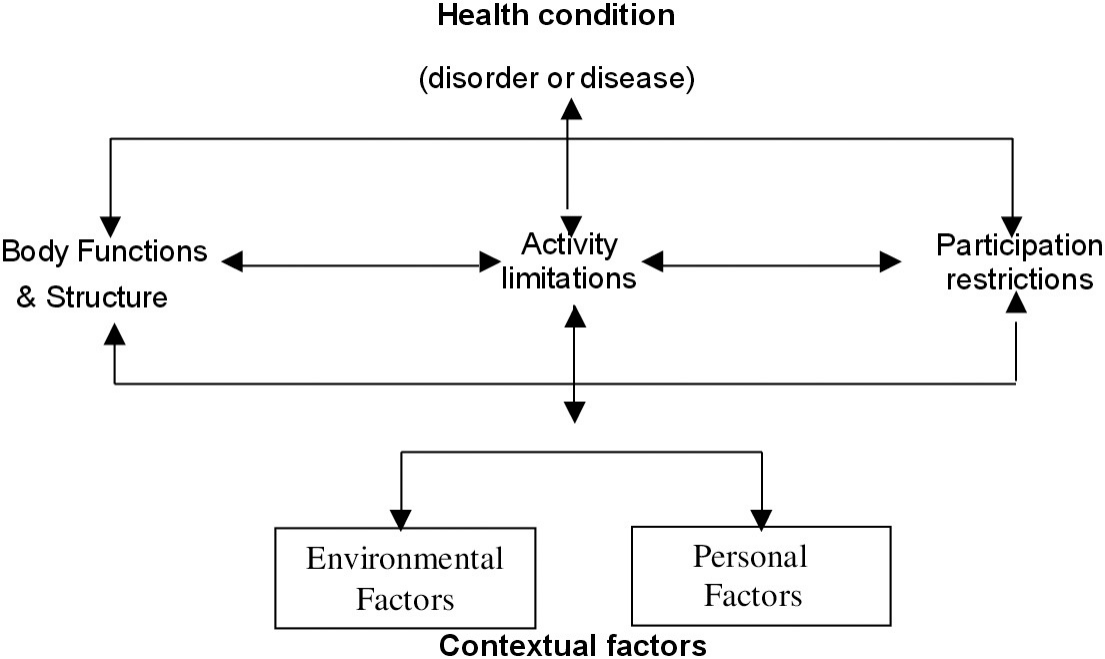
The ICF-WHO framework of disability.

The International Classification of Functioning, Disability, and Health (ICF)-WHO describes disability as dynamic, complex, and multidimensional and defines disability as an umbrella term that covers body impairments, activity limitations, and participation restrictions, resulting in a negative interaction with personal and environmental factors, subsequently leading to disability (Figure 1). The primary emphasis of the new ICF-WHO definition is on the environmental and social factors related to the care needs of PwDs. These environmental factors include assistive products and technology, the natural and built environment, and support from and relationships with other people along with attitudes toward PwDs, services, and systems: governments, organizations, laws, regulations, communication, transportation or cultural systems, and policies (20). Personal factors, however, are not part of the health condition; they indicate the particular background of an individual's life and overall behavioral pattern and character. Personal factors also reflect the individual's motivation and self-esteem (26).

## Preventive Strategies Against COVID-19 Based on the ICF-WHO Disability Framework

As of September 29, 2020, a total of 235 countries or territories, including India, have been affected by SARS-CoV-2, and more than one-third of the global population are in lockdown as part of a mitigation strategy for COVID-19 (3). In such an emergency crisis, PwDs will have to follow restrictive and protective measures taken up by their respective governments. However, many of these strategies recommended by the government will pose challenges to PwDs, as they may face limitations in practicing the protective guidelines. For example, social distancing may not be possible as PwDs are frequently dependent on others, and disruption of the transportation system, because of the lockdown, may affect caregiver's or personal assistant's movement, leading to serious damage or even death for PwDs (13). In the United Kingdom, people with intellectual disabilities and autism face great difficulty in adjusting to these new environments because of the disruption of their daily support system (27).

**Figure 2:**
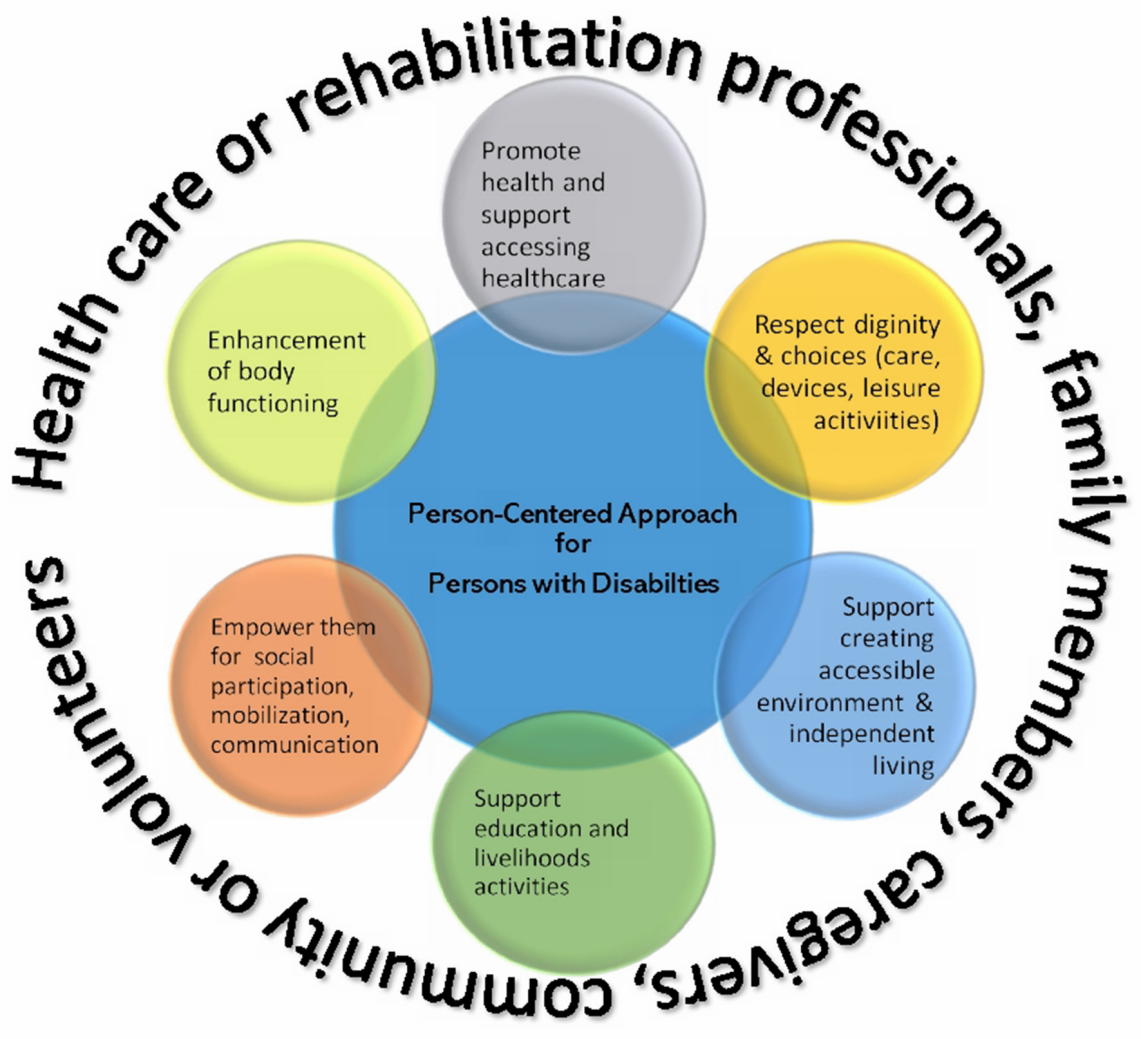
The Person-Centered Approach for people with disabilities.

Disability is a complex, diverse, and growing global concern. There are many chronic health conditions that can lead to a disability. Every person with a disability has unique features and requirements in their lives. Even individuals with the same disability (impaired vision for example), may have different needs according to their age, gender, experiences, education, and other environmental factors. This shows that there is a need to have an Individual Rehabilitation Plan in place for PwDs. In Person-Centered Approaches (PCA), the first and foremost priority is that healthcare or rehabilitation professionals become acquainted with the needs of PwDs. It is not possible to plan a PCA without being aware of the needs or requirements of PwDs. Therefore, the approach will remain a hypothetical construct until the problems faced by PwDs are established. Once their needs have been established, the PCA can then be applied. A few case studies are included as in this article. The elements described in Figure 2 of the PCA may not be applied to every client, therefore, the PCA is a universal framework that can provide practical guidance when planning for disability intervention packages.

In a situation like a national emergency lockdown, development of support systems and provision of support from health-care providers to ensure the continuum of care to PwDs are overarchingly important. These unexpected challenges can potentially be managed through innovative approaches that are conceptualized based on the ICF-WHO disability framework, at the same time employing the principles of PCA. The ICF framework provides aspects or elements that should be considered when planning Person-Centered Approaches.

In a PCA (Figure 2), the individual with a disability is the active participant; family members, caregivers, volunteers community members to a large extent, and health-care providers are the core groups; and they all work together, thereby making a consensus decision to identify the needs of the disabled individual during the lockdown and to formulate the best possible management plan accordingly, while maintaining the disabled individual's dignity, values, and respect. Therefore, in the context of an emergency lockdown, a PCA has the potential to reduce the chance of infection or morbidity and to improve the overall impact on PwDs. The focus of the PCA strategy can be planned according to contextual factors (environmental and personal) and participation restrictions of the ICF-WHO framework (Figure 2). For example, recommendations based on personal factors may include personal behavior changes like wearing a face mask or frequent handwashing.

Since face-to-face interaction between health-care providers and PwDs or other active core members in PCA is not feasible during lockdown, an alternative strategy to educate PwDs, providing them with all the necessary information, can be done through either telehealth or teleconference methods. Videoconferencing is a preferable technique and would be more effective than other modes of communication. The health care team should take the lead in establishing such facilities. They should contact PwDs as well as the core members identified in the approach, and together devise a plan for inclusive services, which are considered appropriate for the best management of PwDs in the time of a pandemic. A special communication platform such as a website for care of PwD during the COVID-19 pandemic can be constructed. This website should link to other health care resources, e.g., emergency contact numbers and phone numbers of local service agencies for essential needs. This study suggests the following three key important areas where such mitigating strategies can be considered.

### Participation Restrictions

Social distancing is an important measure in preventing infection. In the emergency lockdown, PwDs are encouraged to restrict all outdoor movements, and to stay at home. They can be educated about their higher risk of becoming infected and the possible serious illness that may follow if they contract the virus.

Often, PwDs rely on caretakers or caregivers to perform activities of daily living, e.g., bathing, eating, cooking, or doing laundry. These routine tasks can be managed either with a caregiver or by family members, or even by volunteers from the community who participate in planning for person-centered care. During the lockdown, the government can provide special permission to allow caregivers (if any) to commute. The best practice, however, is to have the caregiver stay with the PwD during the lockdown period. The government can also consider launching smartphone applications that can connect users (e.g., PwDs) to a random volunteer who registers in the application, either through video or audio calls. For example, the “Be My Eyes” application helps individuals with visual impairments connect with the first available volunteer (sighted) who registers in the application through video calls, thereby assisting the disabled person with any emergency needs (28).

### Personal Factors

PwDs need to follow various personal protective measures that are helpful in preventing the transmission of the virus. Despite the many challenges present, PwDs can be motivated to follow the recommended guidelines during the pandemic and to apply self-effort to improve their awareness of COVID-19. They need to adopt various new behavioral changes, such as wearing a face mask and maintaining good personal hygiene and sanitation to the maximum possible level. Education and counseling can also be done to improve their self-esteem while ensuring their dignity and preferences remain intact.

### Environmental Factors

Article 25 of the UN Convention on the Rights of Persons with Disabilities reinforces the right of PwDs to achieve the highest possible standard of health and well-being without any form of discrimination based on disabilities (29). Therefore, PwDs' needs should not be ignored during an emergency lockdown. A few recommendations are proposed when responding in the pandemic. First, prepare safe and accessible formats for information on COVID-19, e.g., Braille and sign languages. Second, create an enabling environment for caregivers/peers/community members who can assist disabled persons by providing essential services. Third, provide a supportive environment to meet the daily living requirement within the context of choices made by the disabled person. Fourth, assist in the access for health care services and personal protective equipment. Fifth, improve accessibility of the physical environment. Sixth, promote awareness and sensitization to health-care providers to provide equal opportunities, maintaining dignity and respect whenever a disabled person requires care in hospital. Finally, provide financial support to PwDs during the lockdown period.

### Case Studies of PCA During the Pandemic Lockdown

In India, a sizeable number of young persons with disabilities live in accommodation facilities provided by institutions like schools for the blind, vocational training centers, or stand-alone hostels for disabled persons. When the sudden lockdown was announced those staying in these hostel facilities were impacted significantly as teachers and staff were suddenly absent.

Moreover, many visually challenged people who came to Delhi for a new disability certificate or for renewal of the existing certificate, or vocational training, could not find a place to stay nor were they able to return to their home during the sudden emergency lockdown. There are a number of case studies, many that are unique according to the client's needs, which emerged from our Vision Rehabilitation Clinic (VRC) of Dr. Rajendra Prasad Centre for Ophthalmic Sciences, All India Institute of Medical Sciences, New Delhi that have supported individuals with vision loss through the employment of PCA during the emergency lockdown (30).

#### Case Study 1

A young visually disabled male aged 18 from Azamgarh, Uttar Pradesh, India came to Delhi on April 15, 2020 to renew his disability certificate. Due to the sudden lockdown, he could not find accommodation nor could he return home. He contacted our team and shared his whereabouts. He was filled with panic and anxiety and explained his problems. He was in immediate need of accommodation. Without delay, our VRC team identified the nearest center providing services to visually disabled persons, and contacted the General Secretary of the center, Gurgaon, Haryana. The center later provided him with free accommodation. The team then further communicated with his family members about the situation. The client was educated about protective measures and provided with the appropriate information about the COVID-19 pandemic to avoid any potential misinformation and misconceptions that can aggravate his fear and anxiety. Psychological counseling was also provided.

#### Case Study 2

When the lockdown was relaxed in a phase-wise manner, and transportation was re-opened, visually challenged students who were living in the hostel facilities of schools for the blind in Delhi were asked to leave the hostel and returned to their native homes. Once they reached to their respective villages, the local authorities asked them to stay in the village quarantine facilities which was not accessible for PwDs.

A student male who is 100% visually impaired, aged 16 years, and study in the 9th standard at the Institute for the Blind Amar Colony, Lajpat Nagar, Delhi, was asked to leave the school's hostel facilities by the school's authorities. He left the school on June 8, 2020 and reached his native home village located in Dhanbad, Jharkhand, India on June 10, 2020. Upon reaching his village, he was initially denied home isolation. Our rehabilitation team coordinated with his parents and local leaders or authorities and enquired whether the quarantine facility was accessible for the blind. If not, visually challenged students should be allowed to isolate at his own home. Later he was permitted to isolate in his home. Family members and students with disabilities were educated about COVID-19, including the various protective measures. The student in this case study was also educated about precautionary measures he should have taken during the train journey and before he left hostel.

## Conclusions

The current COVID-19 pandemic which was followed with nationwide emergency lockdowns in many countries worldwide, posing immense challenges to the lives of PwDs. A sudden disruption of support systems can have a serious impact on the health of PwDs and may even endanger their lives. These serious impacts can be minimized with an inclusive approach and planning that is aligned with the principles of PCA, involving PwDs, caregivers, family members, the community to a large extent, and healthcare providers. Once insight and understanding of PCA is gained, planning can move forward according to the needs of PwDs. This article aims to assist healthcare and rehabilitation professionals in constructing an inclusive care plan for PwDs during the pandemic and lockdowns, maybe even during the post-pandemic period. In PCA, PwDs are an active partner, thereafter, the core team can prepare the roadmap. This pandemic also provides an opportunity for health care planners and policymakers in the health sector to implement reforms to ensure disability inclusiveness. The potential impact due to lockdowns can be mitigated if an appropriate planning and policy are in place.

## Data Availability Statement

The original contributions presented in the study are included in the article/supplementary material, further inquiries can be directed to the corresponding author/s.

## Author Contributions

The author confirms being the sole contributor of this work and has approved it for publication.

## Conflict of Interest

The author declares that the research was conducted in the absence of any commercial or financial relationships that could be construed as a potential conflict of interest.
